# Successful Management of *Candida auris* Bloodstream Infection in an Elderly Patient: A Case Report

**DOI:** 10.1002/ccr3.72048

**Published:** 2026-02-23

**Authors:** Arif Hasan Chowdhury, Tania Rahman, Nazrul Islam, Tuhin Sadique, Asif Reza Khan, Debashis Sen, Ahmed Abdullah, Dinesh Mondal, Dilruba Ahmed

**Affiliations:** ^1^ Clinical Microbiology and Immunology Laboratory, Office of Executive Director (OED) icddr,b Dhaka Bangladesh; ^2^ University of Virginia Charlottesville Virginia USA

**Keywords:** global health, health informatics, healthcare management, infectious disease, public health

## Abstract

*Candida auris* is an emerging multidrug‐resistant pathogen frequently misidentified by conventional methods, posing diagnostic and infection‐control challenges, particularly in low‐ and middle‐income countries. We report a 74‐year‐old male in Dhaka, Bangladesh, with multiple comorbidities who developed Candidemia. Blood culture yielded Gram‐positive budding yeast with inconclusive conventional carbohydrate tests. The isolate was identified as *C. auris* using Vitek 2 (software v8.01) and confirmed by ITS sequencing (GenBank accession MZ323988). Phylogenetic analysis showed clustering with global strains. Antifungal susceptibility testing indicated resistance to fluconazole and amphotericin B, with susceptibility to echinocandins. Intravenous micafungin was started based on susceptibility results and guideline recommendations. The patient responded gradually to the treatment, recovered fully, and was discharged after 21 days of admission. This case highlights the importance of rapid species‐level identification and access to confirmatory molecular testing to guide effective treatment and infection prevention in resource‐limited settings.

## Key Clinical Message

*Candida auris* is an emerging multidrug‐resistant pathogen often misidentified as other yeast types. Accurate identification of *C. auris* remains challenging in routine laboratories. This case demonstrates how rapid species‐level diagnosis combined with targeted antifungal therapy can ensure recovery, even in elderly patients with multiple comorbidities.

**Abbreviations**
AST: antifungal susceptibility testing
CDC: Centers for Disease Control and Prevention
ESR: erythrocyte sedimentation rate
ICU: intensive care unit
IDSA: Infectious Diseases Society of America
ITS: internal transcribed spacer
MALDI‐TOF: matrix‐assisted laser desorption/ionization time‐of‐flight
MODS: multiple organ dysfunction syndrome
MSA: multiple sequence alignment
PCI: percutaneous coronary intervention
PCR: polymerase chain reaction

## 1 Introduction

*Candida auris* is an emerging fungal agent that causes invasive infection and is characterized by rising resistance to multiple antifungal agents, along with significant morbidity and mortality [1]. *C. auris* causes infection mainly in patients with underlying morbidities and those who have had healthcare exposure [2]. Identifying the species correctly is essential to prevent its spread and outbreak in hospitals. Identification is also essential to render definitive treatment to patients. *C. auris* is often misidentified as other yeast types unless specific laboratory methods are used [3]. The first study of *C. auris* in Bangladesh revealed the presence of this pathogen in pediatric and adult ICU patients [4]. We report a case of bloodstream infection by *C. auris* in an elderly ICU patient in Bangladesh, confirmed by Vitek 2 and ITS sequencing with phylogenetic analysis, and successfully treated with micafungin, highlighting practical diagnostic and treatment pathways in a resource‐limited context.

## 2 Case History/Examination

On 23rd July 2020, a male patient aged 74 was admitted to a local hospital in Dhaka, Bangladesh with an altered level of consciousness and fever. He suffered a similar attack in the past. Due to the deterioration of his physical condition, he was referred to another hospital with ICU facilities on 30th July.

The patient had chronic kidney disease, cardiovascular disease, diabetes mellitus, and hypothyroidism. In the past, he underwent percutaneous coronary intervention (PCI) for ischemic heart disease. On physical examination, the patient was found on ventilation with sedative and had oxygen saturation (SO_2_) of 99%, respiratory rate of 20 breaths/min, and heart rate of 68 beats/min.

Laboratory investigations showed a platelet count of 457 × 10^3^/μL with 79.8% neutrophils, 11.3% lymphocytes, 3.3% eosinophils, and 5.3% monocytes. Blood hemoglobin was 12.6 g/dL, the erythrocyte sedimentation rate (ESR) was 75 mm/1st hour, serum creatinine was 3.6 mg/dL, C‐reactive protein was 34.8 mg/L with serum electrolyte imbalance (sodium—142 mmol/L, potassium—2.3 mmol/L, chloride—110 mmol/L). Plenty of pus cells and 2+ albumins were detected in routine urine microscopic examination. Urine culture identified *Klebsiella* spp. resistant to amoxicillin, azithromycin, and cephradine.

Abdominal ultrasonography revealed cholelithiasis, bilateral renal cortical cysts, and bilateral renal calculi. Chest X‐ray revealed bilateral pneumonitis. The key clinical events during hospitalization are summarized in Table 1.

**TABLE 1:** Summarized clinical timeline of the patient's hospitalization showing key events.

Date/Hospital day	Key clinical events
23 July 2020	Admitted to a local hospital with fever and altered level of consciousness
30 July 2020	Transferred to a tertiary care hospital due to clinical deterioration
Day 2	Blood collected and sent for culture to the Clinical Microbiology Laboratory of icddr,b; diagnostic evaluation initiated
Day 3–4	Blood culture positive; yeast observed; phenotypic tests inconclusive
Day 4–5	Vitek 2 identified *Candida auris* (99%); AST performed
Day 5	Developed multiple organ dysfunction syndrome (MODS) and underwent tracheostomy to facilitate ventilation
Day 6	Antifungal therapy started with intravenous micafungin
Day 15	Follow‐up blood cultures negative for fungal growth
Day 21	Discharged in stable condition

## 3 Differential Diagnosis

**FIGURE 1:**
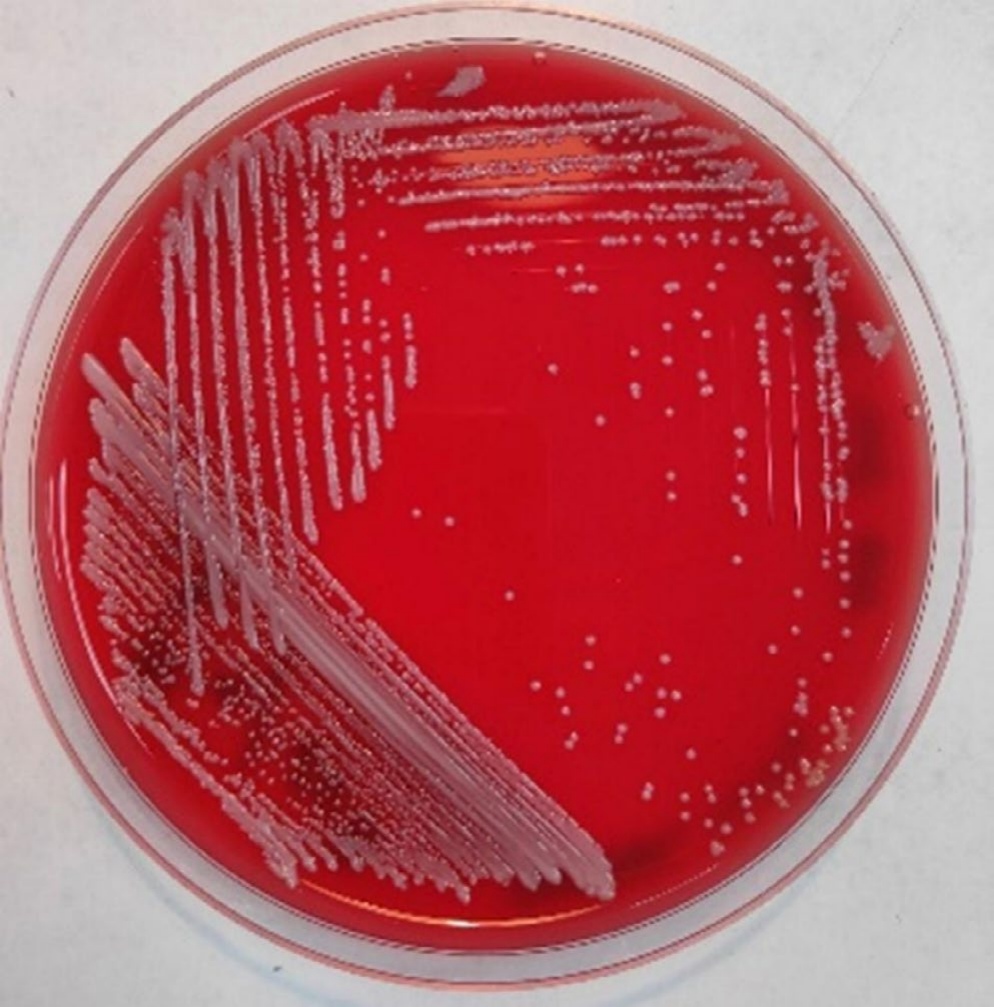
Tiny white opaque, creamy nonhemolytic colonies on 5% Sheep Blood Agar.

**FIGURE 2:**
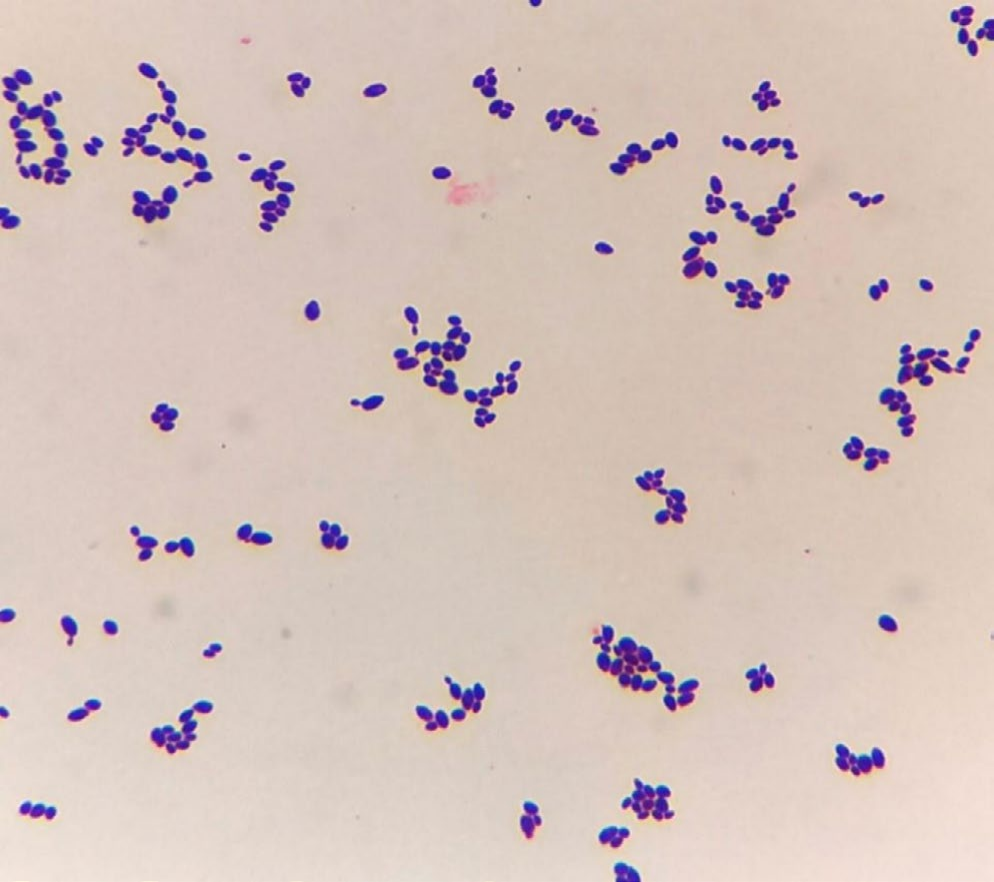
Gram‐stained smear from positive blood culture showing Gram‐positive budding yeast cells.

Blood culture was performed on BacT ALERT which signaled positive. The blood was subcultured on Blood agar, Chocolate agar, and MacConkey agar plates. After overnight incubation, tiny, white, opaque, creamy, nonhemolytic colonies were observed on a 5% Sheep Blood Agar plate (Figure 1); white, creamy colonies were observed on a Chocolate Agar plate; and tiny colonies grew on a MacConkey Agar plate. Gram staining of the culture revealed Gram‐positive budding yeast cells (Figure 2). *Candida* spp. was initially identified using carbohydrate fermentation and assimilation tests. Phenotypic test results are summarized in Table 2.

**TABLE 2:** Phenotypic characteristics of *Candida auris*, isolated from blood sample.

Bacteriological tests performed	Test results
Gram stain	Gram‐positive budding yeast cell
Sugar fermentation	
a. Glucose	Fermented
b. Maltose	Non‐fermented
c. Lactose	Non‐fermented
d. Sucrose	Non‐fermented
e. Galactose	Non‐fermented
f. Trehalose	Non‐fermented
Culture media type	
5% Sheep blood agar	Tiny white opaque, creamy, nonhemolytic colony
Growth temperature	35°C
Vitek 2 (bio number)	6110145265321771

Further identification was performed using the Vitek 2 system, following the manufacturer's guidelines and the latest software update (version 8.01). Based on microscopic examination, a Vitek 2 YST card was used. The Vitek 2 identified the organism as *C. auris* with 99% confidence. Neither CLSI nor EUCAST has recommended specific drugs for *C. auris* . However, for other *Candida* species, Vitek 2 provided antimicrobial susceptibility testing (AST) using the AST card YS08. The isolate demonstrated susceptibility to caspofungin, flucytosine, and micafungin, while showing resistance to amphotericin B and fluconazole.

For molecular confirmation of the pathogen, we extracted genomic DNA from pure culture according to the protocol described elsewhere [5]. PCR and sequence analysis were performed for the ITS 5F (GGAAGTAAAAGTCGTAACAAGG) and ITS 5R (TCCTCCGCTTATTGATATGC) regions [6]. The sequence was submitted to GenBank under accession number MZ323988. Furthermore, we performed phylogenetic analysis to determine the genetic relationships of the studied *C. auris* isolate (MZ323988 CMIL 01) with other global *C. auris* sequences from the GenBank database. The MSA was performed using MAFFT (version 7.480) (https://mafft.cbrc.jp/alignment/software/) with the L‐INS‐i strategy. Default settings were used for other options. A phylogenetic tree was constructed using PhyML (version 3.0) [7]. Model selections were performed using SMS (Smart Model Selection in PhyML). A phylogenetic tree was inferred under the HKY85 + gamma model (Gamma shape parameter: 618.416).

## 4 Conclusion and Results (Outcome and Follow‐Up)

At admission, the patient was started on broad‐spectrum antibacterial therapy with intravenous ceftriaxone, later escalated to meropenem and vancomycin due to clinical deterioration and suspicion of secondary bacterial infection. Following blood culture confirmation of *C. auris* , antifungal therapy with intravenous micafungin (100 mg) was initiated and continued for 14 days. Micafungin was selected because echinocandins are recommended as initial therapy for *C. auris* infections, and echinocandins can be used without renal dose adjustment, unlike amphotericin B, which is associated with clinically significant nephrotoxicity [8, 9, 10]. Combination therapy was not used because the patient responded well to echinocandin monotherapy, and amphotericin B and fluconazole were not suitable options due to resistance.

During the ICU stay, the patient received supportive care with close monitoring of fluid balance and electrolytes. Follow‐up blood cultures taken on Day 15 were negative for fungal growth, and inflammatory markers steadily declined. His condition gradually improved, and after 21 days in the hospital, he was discharged in stable condition without signs of persistent bloodstream infection.

## 5 Discussion

**FIGURE 3:**
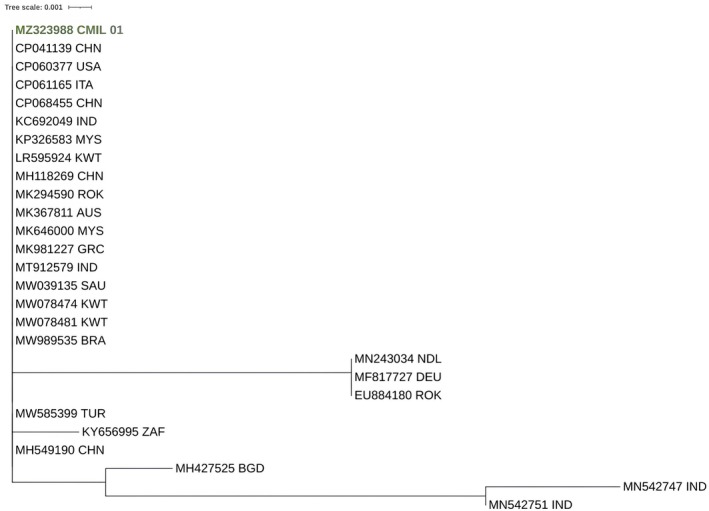
Phylogenetic analysis of *Candida auris* isolate (MZ323988; CMIL 01) based on ITS sequencing. The Bangladeshi isolate clusters with global strains reported from Asia and other regions, supporting its placement within internationally circulating clades and highlighting the global spread of *C. auris* .

This case demonstrates that *C. auris* candidemia can be successfully identified and treated in a resource‐limited setting when clinicians maintain a high index of suspicion and laboratories prioritize species‐level confirmation. Precise species‐level identification is crucial for detecting *C. auris* and implementing proper infection prevention strategies [11]. Clinical laboratories should be aware of, and alert to, the potential for misidentification of *Candida auris* when using phenotypic methods such as manual biochemical testing, API 20C, Phoenix (BD), MicroScan (Beckman Coulter, Pasadena, CA), and Vitek 2 (bioMérieux). We used Vitek 2 with software version 8.01 and accurately identified *C. auris* , although studies have reported misidentifications of *C. auris* strains from certain clades [12]. As *C. auris* continues to gain worldwide attention, laboratories should consult their instrument manufacturers to update their software versions that include *C. auris* in their yeast identification cards. Diagnostics based on MALDI‐TOF have shown higher specificity in differentiating *C. auris* from other species. Molecular techniques, including PCR amplification and sequencing of the Internal Transcribed Spacer (ITS) region of rDNA, remain the standard method for identifying *C. auris* at the species level [12]. The phylogenetic tree generated from the gene sequences of *C. auris* isolates (Figure 3) demonstrated that our strain was present in the clade of strains from China, the USA, Italy, India, Malaysia, Greece, Kuwait, and Brazil.

Global surveillance studies continue to demonstrate the spread of multiple *C. auris* clades across continents. Our phylogenetic analysis showed clustering of the Bangladeshi isolate with strains from Asia, Europe, and North America, highlighting the international circulation of this pathogen. Recent genomic studies have also identified the emergence of a sixth clade, underscoring the ongoing evolution of *C. auris* and the need for regional sequencing data [13].

Therapeutically, echinocandins remain the recommended first‐line agents for candidemia due to *C. auris* , as supported by CDC and IDSA guidelines [8]. However, resistance to echinocandins was reported, particularly in isolates carrying FKS1 mutations [14], Amphotericin B resistance is common, while azole resistance, especially to fluconazole, approaches 90% in some regions [15]. This underscores the urgent need for antifungal stewardship, development of new agents, and routine antifungal susceptibility testing.

A key lesson from this case is the need for diagnostic vigilance when routine phenotypic yeast identification is inconclusive. In our case, only glucose was fermented, while all other carbohydrates were neither fermented nor assimilated. This raised suspicion of an uncommon *Candida* species. Identification using Vitek 2 (software version 8.01) provided high‐confidence detection of *C. auris* , which was subsequently confirmed molecularly via ITS sequencing. This stepwise approach illustrates a practical pathway for laboratories in resource‐limited settings: recognize atypical biochemical patterns, escalate to automated identification systems with updated databases, and confirm using molecular methods when possible.

Beyond individual patient management, *C. auris* has major infection‐control implications due to its ability to persist on surfaces, cause nosocomial transmission, and lead to hospital outbreaks [8, 12]. Species‐level identification should therefore trigger immediate infection prevention actions. From a public health perspective, both confirmed and suspected *C. auris* infections should be promptly reported to surveillance systems and public health laboratories, as coordinated efforts involving clinical laboratories, antimicrobial stewardship programs, and infection‐control teams are critical to limit institutional transmission [8]. In settings such as Bangladesh and similar low‐ and middle‐income countries, strengthening laboratory capacity, reporting pathways, and clinical awareness is essential to prevent delayed recognition and minimize the risk of healthcare‐associated spread.

Limitations of this report should be acknowledged. As a single‐patient case, the findings have limited generalizability. Therapeutic drug monitoring of antifungal agents was not performed. In addition, screening for colonization among contacts and environmental sampling were not undertaken, which would have provided valuable insight into potential transmission dynamics. Despite these constraints, this case highlights the importance of diagnostic vigilance: inconclusive biochemical testing prompted suspicion of an uncommon species, and timely species‐level identification followed by antifungal susceptibility testing (AST)–guided therapy contributed to clinical recovery while supporting infection prevention measures in a resource‐limited healthcare setting.

## 6 Conclusion

This case highlights the clinical significance of *C. auris* bloodstream infection in a patient with multiple risk factors and demonstrates the importance of accurate laboratory identification, molecular confirmation, and targeted antifungal therapy. Timely initiation of appropriate treatment contributed to a favorable outcome, despite the severity of illness and underlying comorbidities. Increased awareness among clinicians, access to reliable diagnostic tools, and careful antifungal stewardship are critical to reducing the risk of misidentification, improving patient outcomes, and preventing nosocomial transmission of this emerging pathogen.

## Author Contributions

**Arif Hasan Chowdhury:** conceptualization, investigation, resources, writing – review and editing. **Tania Rahman:** formal analysis, methodology. **Nazrul Islam:** conceptualization, investigation, supervision, validation. **Tuhin Sadique:** conceptualization, data curation, methodology, writing – review and editing. **Asif Reza Khan:** data curation, investigation, writing – original draft, writing – review and editing. **Debashis Sen:** formal analysis, software, writing – review and editing. **Ahmed Abdullah:** formal analysis, investigation. **Dinesh Mondal:** investigation, validation, writing – review and editing. **Dilruba Ahmed:** conceptualization, data curation, formal analysis, writing – review and editing.

## Funding

The authors have nothing to report.

## Ethics Statement

Ethical approval was not required for this case report in accordance with local guidelines. The study was conducted in accordance with the Declaration of Helsinki.

## Consent

Written and informed consent was obtained from the patient to publish this case report and any accompanying images.

## Conflicts of Interest

The authors declare no conflicts of interest.

## Data Availability

Data sharing is not applicable to this article as no datasets were generated or analyzed during the current study.
